# Safety and efficacy of total thoracoscopic surgery for patients with tricuspid regurgitation and reduced right ventricular function after left heart valves replacement: a retrospective comparative study

**DOI:** 10.1186/s12872-023-03428-z

**Published:** 2023-08-14

**Authors:** Zhiqin Lin, Zheng Xu, Xiujun Chen, Feng Lin, Liangwan Chen, Xiaofu Dai

**Affiliations:** 1 Department of Cardiovascular Surgery, Union Hospital, Fujian Medical University, Xinquan Road 29#, Fuzhou, 350001 P. R. China; 2 Key Laboratory of Cardio-Thoracic Surgery, Fujian Medical University, Fujian Province University, Fuzhou, 350001 P. R. China

**Keywords:** Total thoracoscopic tricuspid valve replacement, Tricuspid valve replacement, Tricuspid regurgitation, Right ventricular function

## Abstract

**Background:**

Tricuspid valve surgery is the standard treatment for tricuspid valve disease refractory to pharmacologic therapy. However, patients with tricuspid regurgitation after previous left heart valves replacement with reduced right ventricular (RV) function are at greater risk of surgery. We compared the clinical outcomes of tricuspid valve replacement in this subgroup of patients through mini-thoracotomy and conventional full-sternotomy approach.

**Methods:**

We identified 44 patients at our institution with tricuspid regurgitation and reduced right ventricular function after left heart valves replacement who underwent either total thoracoscopic tricuspid valve replacement (T-TVR) or conventional tricuspid valve replacement (C-TVR) from December 2014 and May 2021. Patient clinical characteristics, hospital course, and postoperative changes in RV function were retrospectively reviewed and analyzed.

**Results:**

Baseline characteristics between T-TVR (*n* = 25) and C-TVR (*n* = 19) were comparable including a high incidence of liver dysfunction and renal insufficiency. There were no statistically differences between the two groups in terms of hospital mortality (8.0% vs. 21.1%, *P* = .211). Patients in the T-TVR group had less total drainage volume (201.60 ± 77.05 ml vs. 614.74 ± 182.31 ml, *p* < .001), required fewer postoperative blood product transfusions, and had a lower total length of hospital stay (15(15–16) vs. 16(14–17) days, *P* = .019) compared to the C-TVR group. T-TVR was associated with better and faster recovery of tricuspid annular plane systolic excursion (TAPSE) and right ventricle fractional area change (RVFAC) (adjusted β = 0.154, 95% CI: 0.037 to 0.271, *p* = .010 and adjusted β = 0.003, 95% CI: 0.000 to 0.005, *p* = .024; respectively) within the first 3 months postoperatively compared with C-TVR.

**Conclusions:**

T-TVR represents a viable alternative to current surgical strategies as a potentially sicker cohort demonstrated similar hospital mortality compared to conventional surgery, with reduced length of hospital stay, fewer blood transfusions, and more favorable in promoting RV functional recovery in the early period. Future prospective, randomized-controlled trials with longer follow-up durations are needed to validate these findings.

## Background

Tricuspid regurgitation (TR) and right ventricular (RV) dysfunction are associated with delayed outcome after left-sided valve surgery. [1, 2] Tricuspid valve replacement (TVR) in the later stages of RV dysfunction is still a very controversial issue when considering the indications for surgery and surgical technique [3]. Reoperative cardiac surgery through a median full-sternotomy in these patients is technically challenging, because of a thin right atrial wall and dense pericardial adhesions, increasing the risk of intraoperative and postoperative bleeding. Additionally, many of these patients represent a high-risk population with a long medical history and severe comorbidities.

In recent years, the use of minimally invasive thoracoscopic techniques in cardiac surgery has been shown to improve procedural safety and clinical outcome, [4] which may be an alternative for high-risk patients who indicated for conventional TVR (C-TVR) through a median full-sternotomy in the late stage after left valve surgery. In re-performing cardiac surgery, minimally invasive thoracoscopic surgery can reduce intraoperative risk by avoiding damage to adjacent cardiac structures and extensive mediastinal dissection. However, there is insufficient data on whether patients with tricuspid valve lesions combined with reduced RV function after left heart surgery can benefit from minimally invasive thoracoscopic surgery. The purpose of this study was to assess the postoperative clinical outcomes after total thoracoscopic TVR (T-TVR) in patients with TR presenting after left heart surgery accompanied by reduced right ventricular function and to compare the perioperative outcomes with patients undergoing C-TVR.

## Methods

### ***Study patients and data collection***

We conducted a retrospective study of 44 consecutive patients with severe TR and reduced RV function who underwent isolated TVR (T-TVR or C-TVR) after previous sternotomy left heart surgery at our institution between December 2014 and May 2021. The study population was identified from the hospital’s electronic health records using specific diagnostic and procedural codes related to TVR. Patients were included in the study if they met the following criteria: (1) Age ≥ 18 years; (2) Diagnosis of severe functional TR due to annular dilatation and/or leaflet restriction; (3) Reduced RV function, defined as patients presenting with varying degrees of signs of right-sided heart failure (e.g., lower extremity edema, ascites, pleural effusion, jugular vein dilatation, congestive renal failure) and echocardiographic findings of tricuspid annular plane systolic excursion (TAPSE) less than 16 mm or right ventricle fractional area change (RVFAC) less than 35% [5]; (4) History of previous sternotomy left heart surgery; (5) Underwent isolated TVR, defined as TVR with no concomitant cardiac surgery during reoperation (e.g., mitral or aortic replacement, radiofrequency ablation of atrial fibrillation, or coronary artery bypass grafting).The preoperative RV dysfunction severity was depended on the attending who carefully conducted a consultation and physical examination, and reviewed the echocardiography.

#### Data collection process

Clinical, demographic, and echocardiographic data were collected retrospectively from electronic health records using a standardized data collection form. All available medical and surgical records were reviewed for each patient, including admission notes, progress notes, discharge summaries, laboratory results, and imaging studies. Data were abstracted by two independent researchers, and any discrepancies were resolved through discussion and consensus. To ensure the accuracy and completeness of our data, we performed telephone interviews with patients or their caregivers, if needed, to obtain information on clinical variables that were not available in the electronic health records.

The primary outcomes of interest were early major complications that included low cardiac output syndrome (LCOS), requiring mechanical circulatory support, postoperative bleeding, postoperative blood transfusion, prolonged ventilation (> 24 h) and pain intensity. Early mortality was defined as a death occurring within 30 days of surgery or during the same hospitalization after surgery. The secondary outcome of interest was early RV functional recovery. The postoperative drainage volume was recorded daily and the tube was removed when there was < 100 mL per day, usually 2–4 days after operation. Postoperative pain intensity was evaluated by the visual analogue scales (VASs) on the 7th day after the patient’s surgery [6]. Transthoracic echocardiography examinations were routinely performed using a commercially available imaging system equipped with 3.5 MHz transducers (Vivid E9; General Electric Healthcare) preoperatively and postoperatively to grade the size and function of the right ventricle. Postoperative echocardiography was performed during the first and twelfth postoperative week. TR was graded as mild, moderate, or severe defined by a distal jet area of less than 5 cm^2^, 5 to 10 cm^2^, and more than 10 cm^2^, respectively, according to the proximal isovelocity surface area method [7]. Echocardiographic measurements for right ventricle systolic function included TAPSE by M-mode, and RVFAC [8].

The Ethics Committee of Union Hospital, Fujian Medical University, approved our study, and waive informed consent because of the retrospective nature of the present study. No patients were directly involved in the study. All methods were carried out in accordance with relevant guidelines and regulations.

### Surgical procedures

The decision to perform T-TVR and C-TVR was made according to the preference of the heart surgeon at our institution. Patients with thoracic adhesions due to infection, tumor, or previous right-sided thoracotomy were excluded from thoracoscopic surgery because of difficulty in lung mobility. The decision to perform the operation with or without aortic cross-clamping was made by the surgeon based on the intraoperative situation. Patients in the T-TVR group underwent surgery under thoracoscopic guidance through a 1 cm camera incision at the level of the axillary midline and a 3–4 cm main working port of anterolateral mini-thoracotomy in the fourth intercostal space. The patients were placed in the left semi-lateral decubitus with the right chest elevated at 25–30^◦^. Cardiopulmonary bypass (CPB) was performed through the femoral vein, artery, and internal jugular vein. We used vacuum-assist double venous drainage, which is an effective strategy for stabilizing the drainage to maintain a clear surgical field. The pericardium was dissected along the right atrial wall and the tricuspid valve was replaced with heart beat or aortic cross-clamping. Patients in the C-TVR group underwent repeat median sternotomy with an oscillating saw, followed by careful dissection of pericardial adhesions. In cases of severe adhesions leading to hemorrhage, femoral CPB was used to mitigate the risk of aortic or cardiac rupture.

### Statistical analysis

Continuous variables were expressed as the median or mean ± standard deviation and were evaluated using Student’s t-test. Categorical variables are presented as counts or percentages and were evaluated using Fisher’s exact test. Non-normally distributed variables are reported as the median (interquartile range [IQR]) and were evaluated using the Wilcoxon signed-rank test. We performed generalized estimating equations (GEE) to analyze RV functional recovery over time, and to assess whether RV functional recovery differed between the T-TVR and C- TVR groups. To compare changes in TAPSE and RVFAC over time in the T-TVR and C- TVR groups, we included an interaction term (group × time) in the GEE models. Crude and adjusted β coefficients with 95% confidence intervals (CIs) were reported to estimate the strength of the association between surgical strategy and RV functional recovery outcomes. Statistical significance was defined as a two-sided P-value of < 0.05 was considered statistically significant. The statistical software used throughout the analysis was SPSS v.26.0 (IBM SPSS Inc., Armonk, NY) and R 4.0.1.

## Results

### Baseline characteristics

A total of 44 cases were included in this study; 25 patients for T-TVR and 19 patients for C-TVR. Two of the patients in the C-TVR group crossed over from T-TVR to C-TVR owing to extensive adhesion in the right-sided thoracic cavity. Six patients in the T-TVR group and 1 patient in the C-TVR group underwent De Vega’s tricuspid annuloplasty during the first left-sided valve surgery. Baseline characteristics and comorbidities are shown in Table 1. The preoperative baseline characteristics were similar between the two patient groups.

**Table 1:** Comparison of patients’ baseline demographic and clinical characteristics

Variables^a^	Total sample (*n* = 44)	Patient groups		
		Thoracoscopy (*n* = 25)	Sternotomy (*n* = 19)	*P* value
Age (yr)	57.0(51.5–64.0)	57.0(50.0–62.0)	57.0(53.5–65.0)	0.302
Male (n)	25	16	9	0.426
BMI (kg/m2)	20.70(18.65–24.65)	21.81(18.65–24.65)	19.97(18.43–22.95)	0.278
Smoking history (n)	11	7	4	0.598
Diabetes (n)	7	5	2	0.395
Hypertension (n)	20	13	7	0.487
CAD(n)	10	6	4	0.817
Prior MI (n)	2	2	0	0.207
COPD (n)	9	5	2	0.092
liver dysfunction (n)	21	14	7	0.339
Dialysis (n)	4	2	2	0.772
Peripheral Vascular Disease	1	1	0	0.378
Cancer history (n)	4	2	2	0.773
Stroke history (n)	6	4	2	0.600
AF (n)	17	11	6	0.599
Endocarditis	5	3	2	0.878
NYHA class (n)				
II	8	4	4	0.621
III	26	14	12	
IV	10	7	3	
Prior cardiac surgery				
Time to reoperation (year)	21.61 ± 8.37	19.60 ± 8.52	24.26 ± 7.58	0.067
Mitral valve (n)	23	12	11	0.729
Aortic valve (n)	29	18	11	0.511
Tricuspid valve (n)	7	6	1	0.092
Preoperative PPM/AICD	2	2	0	0.207
Echocardiographic data				
LVEF (%)	55.02 ± 5.42	54.17 ± 5.41	56.18 ± 5.38	0.989
Internal diameters of RA (mm)	62.62 ± 8.95	51.51 ± 8.18	64.08 ± 9.92	0.352
SPAP (mmHg)	59.60 (55.80–74.85)	64.80 (58.20–74.60)	57.10 (54.85–75.40)	0.197
Internal diameters of RV (mm)	25.28 ± 2.19	25.06 ± 2.43	25.56 ± 1.86	0.460
TAPSE	10 (9–11)	10 (9–12)	10 (9–11)	0.547
RVFAC (%)	37.9 (36.0–41.8)	39.5 (36.5–41.4)	37.1 (35.1–42.0)	0.255

*Abbreviations*: *BMI* Body mass index, *NYHA* New York Heart Association, *AF* Atrial fibrillation, *COPD* Chronic obstructive pulmonary disease, *CAD* Coronary artery disease, *MI* Myocardial infarction, *PPM* Permanent pacemaker, *AICD* Automatic implantable cardioverter defibrillator, *LVEF* Left ventricular ejection fraction, *LVEDD* Left ventricular end-diastolic dimension, *BAV* Bicuspid aortic valve, tricuspid regurgitation pressure gradient. *SPAP* systolic pulmonary artery pressure, *RVFAC* Right ventricular fractional area change

^a^ Non-normally distributed variables are presented as the median [interquartile range (IQR)] and categorical data as number

### Operative data and early outcomes

Table 2 shows the operative data and early outcomes of the study participants. The duration of operation (258.52 ± 21.46 min versus 203.84 ± 18.11 min, *P*＜0.001) and the time of CPB (99.00 [95.50–109.00] minutes versus 93.00[83.00-103.00] minutes, *P*＜0.001) were significantly higher in the C-TVR group. There was a significant difference in cardiac arrest CPB between the two groups, which was required in three patients in the T-TVR group and eight patients in the C-TVR group (*p* = .022).

**Table 2:** Operative data and postoperative in-hospital outcomes

Variables^a^	Total sample (*n* = 44)	Patient groups		
		Thoracoscopy (*n* = 25)	Sternotomy (*n* = 19)	*P* value
Operation duration (minutes)	227.45 ± 33.57	203.84 ± 18.11	258.52 ± 21.46	< 0.001
CPB Time (minutes)	96.00 (88.75–106.25)	93.00 (83.00–103.00)	99.00 (95.50–109.00)	0.018
Operated with aortic cross-clamping (n)	9	3	8	0.022
Intensive care unit stay (days)	5 (4–6)	5 (4–5)	6 (5–8)	0.013
Hospital stay (days)	17 (16 -18)	15 (15–16)	16 (14–17)	0.019
Hospital mortality	13.6%	8.0%	21.1%	0.211
Bioprosthetic valve implantation (n)	6	3	3	0.657
Mechanical valve implantation (n)		3	2	
Need for NO (n)	7	4	3	0.985
Transfusion				
Total red cell (unit)	3.0 (0.0–5.6)	0.0 (0.0–2.0)	6.0 (4.3–7.0)	< 0.001
Total serum volume (mL)	200.0 (0.0–300.0)	200.0 (0.0–300.0)	200.0 (0.0–300.0)	< 0.001
Platelet (unit)	0.5 (0.0–1.0)	0.0 (0.0–0.4)	0.0 (0.8–1.4)	< 0.001
Early complications	56	21	39	
Respiratory complication (n)	20	10	10	0.598
Prolonged ventilation (n)	12	5	7	0.367
LCOS requiring MCS (n)	4	1	3	0.178
Cardiocerebral events (n)	5	3	2	0.879
Pacemaker implantation (n)	5	3	2	0.879
Dialysis (n)	6	2	4	0.211
Drainage (millilitres)	376.48 ± 167.52	283.40 ± 78.99	498.98 ± 175.65	< 0.001
Re-exploration for bleeding (n)	1	0	1	0.246
Postoperative echocardiographic results ^b^				
LVEF (%)	54.23 ± 5.42	55.04 ± 3.08	53.13 ± 4.54	0.264
Internal diameters of RA (mm)	70.70 (68.05–75.30)	69.95 (68.30–74.95)	70.80 (67.65–75.55)	0.945
Internal diameters of RV	21.65 (19.95–22.60)	21.60 (19.30–23.70)	21.70 (21.05–22.40)	0.393
TAPSE	15 (12–17)	16 (15–17)	12 (10–15)	0.001
RVFAC (%)	53.2 (45.6–56.4)	53.6 (46.2–57.1)	52.4 (43.7–54.8)	0.158
VASs Score	4.0 (2.0–6.0)	3.0 (2.0–4.0)	6.0 (5.0–8.5)	< 0.001

*Abbreviations*: *CPB* Cardiopulmonary bypass, *LCOS* Low cardiac output syndrome, *MCS* Mechanical cardiac support, *LVEF* Left ventricular ejection fraction, *RA* Right atrium, *RV* Right ventricle, *TAPSE* Tricuspid annular plane systolic excursion, *RVFAC* Right ventricular fractional area change, *VAS* Visual analogue scales

^a^ Non-normally distributed variables are presented as the median [interquartile range (IQR)] and categorical data as number

^b^ 1 week after surgery

The C-TVR group had a significantly longer duration of intensive care unit (ICU) stay (6[5,–8] days versus 5[4–5) days, *P* = .013) and a significantly longer duration of hospital stay (16[14,–17] days versus 15[15,–16] days, *P* = .019) than the T-TVR group. T-TVR patients demonstrated an incidence of hospital mortality similar to that of C-TVR patients (T-TVR, 8.0%; C-TVR, 21.1%; *P* = .211). There was significantly less total drainage volume, postoperative red cell transfusion, serum volume and platelet usage for patients in the T-TVR group compared with patients in the C-TVR group (all *P* < .001). Postoperative pain score between T-TVR and C-TVR was significantly different (6[5,–8] versus 5[4,–5], *P* < .001). Postoperative low cardiac output syndrome occurred in three patients in the T-TVR group and five patients in the C-TVR group. One patient from the T-TVR group was treated with an intra-aortic balloon pump and three cases in the C-TVR group were administered with extra-corporal membrane oxygenation. The overall hospital mortality was 13.6% with two (8.0%) deaths occurring in the T-TVR group and four (21.1%) deaths in the C-TVR group (*P* = .211).

### Comparison of changes in TAPSE and RVFAC recovery

**Fig. 1:**
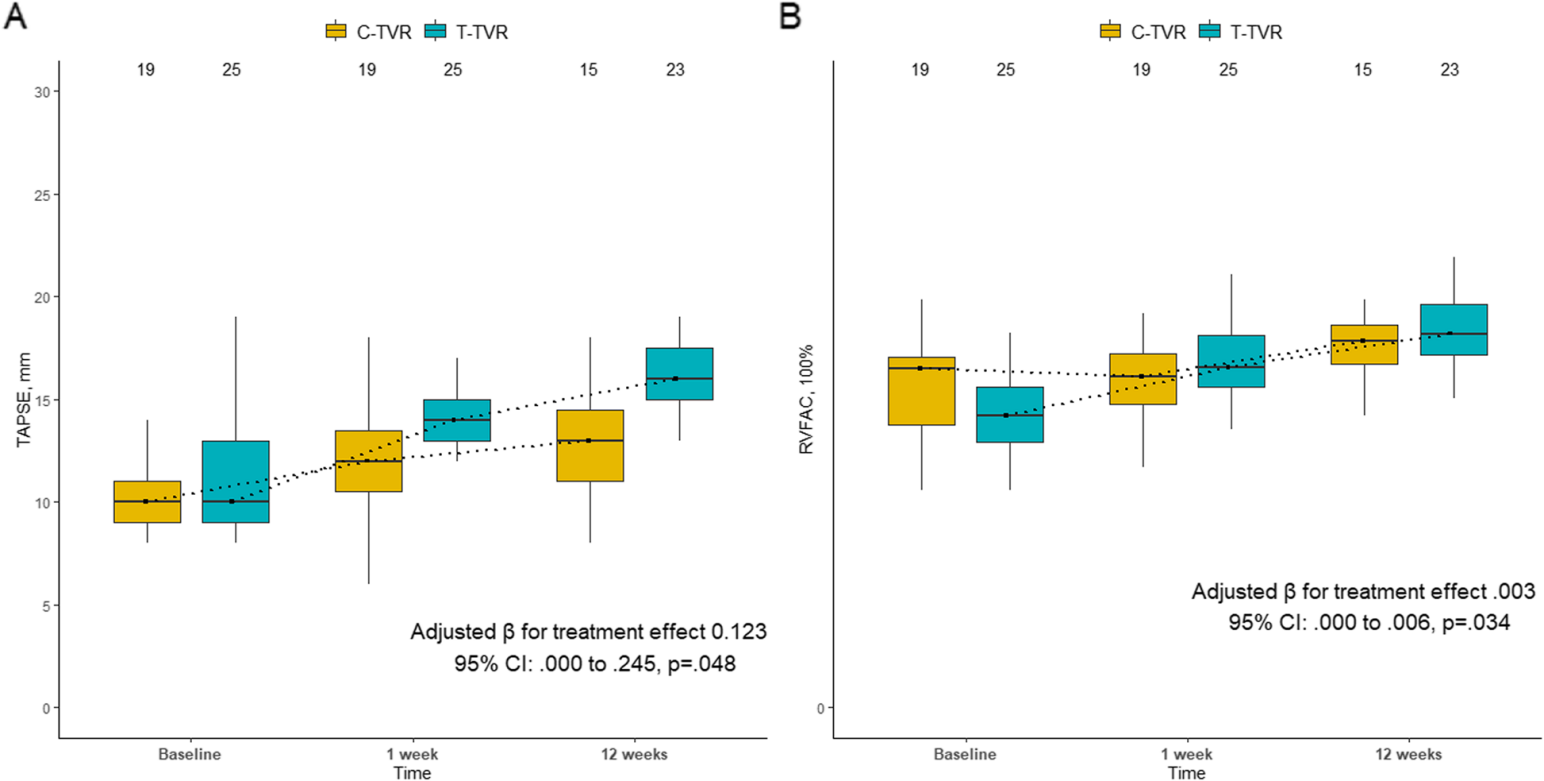
TAPSE and RVFAV of the two groups at baseline and follow-up **A**, Median [interquartile range] of TAPSE of the two groups at baseline and follow-up. **B**, Median [interquartile range] of RVFAV of the two groups at baseline and follow-up. T-TVR, thoracoscopic tricuspid valve replacement; C-TVR, conventional tricuspid valve replacement

Figure 1 A, B shows the results of the groups at baseline, 1week, and 3 months regarding TAPSE and RVFAC over time. GEE analysis showed that surgical strategy (T-TVR or C-TVR) had a significant influence on RV functional recovery. Following adjustment of baseline clinical characteristics, TAPSE at one week (*P* < .001) and three months (*P* < .001) increased over baseline in the T-TVR group and showed greater improvement within the first three months postoperatively (adjusted β for treatment effect 0.154, 95% CI: 0.037 to 0.271, *p* = .010) compared with the C-TVR group. RVFAC at one week (*P* < .001) and three months (*P* < .001) improved over baseline in the T-TVR group and showed greater improvement within the first three months postoperatively (adjusted β = 0.003, 95% CI: 0.000 to 0.005, *p* = .024) compared with the C-TVR group after covariate adjustment.

## Discussion

This study described the early clinical outcomes in patients with reduced RV function undergoing reoperative TVR via total thoracoscopic surgery and full sternotomy approach at a single institution.

Severe functional TR, which is often associated with severe RV dysfunction, has been reported in 27% of patients who underwent previous left-sided valve surgery [9]. Previous studies identified RV dysfunction as a predictor of poor outcome after reoperative TVR. Current guidelines recommend surgery only in patients with severe TR after left-sided surgery and without severe RV dysfunction. [10, 11] Therefore, the decision to perform reoperative TVR in patients with reduced RV function is challenging. We concentrated on this subgroup of patients since we believe this is a high-risk surgical population with poorer clinical condition and had severe comorbidities resulting from systemic venous congestion due to exacerbation of preexisting RV dysfunction.

This group of patients tend to experience more postoperative complications that lead to prolonged intensive care unit (ICU) and total hospital stay, which leads to higher short and long-term mortality. The patients in our study had severe RV dysfunction on admission and immediate surgical intervention was generally inappropriate. We recommend optimizing preoperative drug therapy, particularly strengthening diuresis, to reduce RV volume overload until symptoms of RV dysfunction such as edema, are alleviated.

Our experience in this series of patients with severe RV dysfunction suggests that patients undergoing total thoracoscopic reoperation have better early outcomes, including shorter hospital stays and ICU stays, which are in agreement with of the literature. Previous studies have shown that isolated tricuspid valve surgery is associated with high mortality, particularly in the presence of reoperation and RV dysfunction. In-hospital mortality for the entire cohort in our study was 13.6%, similar to the range of 13–26% reported by other studies, [12] and appears to be quite encouraging when in-hospital mortality in the T-TVR group was 8.0%.

In line with other series involving minimally invasive TV surgery, this study showed that T-TVR seems to have a tremendous advantage over C-TVR in terms of the reducing operative time, drainage amounts, and transfusion requirement [13]. The longer operation time and CPB time in the C-TVR group may be attributed to the presence of scar tissue from previous left heart valve surgery, which increased the difficulty and risk of reoperation. The cardiac arrest CPB was also more frequently needed in the C-TVR group to facilitate exposure and manipulation of the tricuspid valve. Moreover, we excluded patients with thoracic adhesions due to infection, tumor, or previous right-sided thoracotomy from T-TVR, which may have also contributed to the shorter operative time in the T-TVR group. According to Stoppe et al., shorter CPB time would likely lead to shorter ICU and hospital stay durations and faster postoperative recovery [14]. Moreover, less postoperative bleeding and a decreased requirement for transfusion probably indicated more stable hemodynamics, facilitating early removal of the tracheal intubation [15]. The T-TVR group had a shorter operative time and extracorporeal circulation time, which reduced the impact of surgery on postoperative recovery. Patients with relatively intact chest and reduced pain after thoracoscopic surgery avoided sternal infection, and sternal instability, and were able to perform respiratory function exercises and resume daily life activities earlier. It should be emphasized that low postoperative blood loss was also responsible for this difference. Aikebaier et al. reported 49 cases of isolated tricuspid redo procedure, with a median re-sternotomy in 23 patients and a right anterolateral thoracotomy in 26 patients. The results showed that the total drainage amount was lower in the right anterolateral mini-thoracotomy group (*P* = .012) [16]. Gavin et al. determined that blood product transfusion in patients undergoing cardiac surgery is strongly associated with hospital stay, increased early and late mortality, and hospital costs. Minimally invasive thoracoscopic technique can reduce bleeding by avoiding sternal injury and extensive fibrous adhesion separation. In the T-TVR group, it was also observed that there was less postoperative mediastinal drainage and therefore less need for transfusion of blood products, lead to shorter stays in the ICU, and reduced postoperative hospitalization time.

Studies on RV functional recovery in patients undergoing isolated reoperative TVR are scarce. It is important for surgeons to assess the status of RV functional recovery because RV dysfunction is a major independent predictor of all-cause mortality in this subgroup of patients. Assessment of right ventricular functional recovery improves risk stratification in this high-risk group and provides long-term prognostic data. We found a significantly faster recovery of RV function with significant improvement in TAPSE and RVFAC in the T-TVR group. We speculate that this may be related to the shorter operative time, shorter CPB time, and higher possibility of on-pump beating heart surgery in the T-TVR group, which allows for better protection of the RV myocardium during the procedure and facilitates recovery of cardiac function after surgery. Another reason is the reduced risk of potential damage to the heart tissue, especially the right ventricle, from thoracoscopic surgery. In this setting of severe functional TR secondary to left heart surgery, RV dysfunction may be reversible and could recover to a certain extent in the early postoperative period after TVR, which is therefore an advantage; however, determining whether it can lead to full recovery and achieve consistent stability requires further long-term observation.

An increasing number of alternative therapies have been introduced for structural heart diseases. Transcatheter tricuspid valve implantation (TTVI) has also begun to play an important role in tricuspid stenosis and insufficiency. However, TTVI is a new treatment alternative that is still in its infancy, and it is less cost-effective compared to thoracoscopic surgery.

Most surgeons refer to the recommendations for functional tricuspid valve disease in the European Society of Cardiology (ESC)/ European Association for Cardio-Thoracic Surgery (EACTS) guidelines on the management of valvular heart disease (version 2017) guidelines and the American College of Cardiology (ACC)/American Heart Association (AMA) guidelines (version 2006), and choose tricuspid valve repair as the preferred treatment. [17, 18] Reubendra et al. reported that repair of the tricuspid valve is feasible in the majority of patients with functional tricuspid valve disease [19]. However, in another study, Guillaume et al. presented that replacement is more effective in early and late corrections of regurgitation after TV surgery, [20] However, in another study, McCarthy et al. concluded in their research that follow-up echocardiography demonstrated recurrent moderate-to-severe regurgitation in 20% of patients undergoing repair in the long term after surgery [21]. Recurrent TR is a significant problem that may affect the survival and quality of life after tricuspid valve repair. The choice of replacement or repair should be based on the overall state of the patient and personal experience. In our opinion, for patients with severe right ventricular dysfunction, timely and reasonable TVR may be the first choice, which can prevent the occurrence of severe right ventricular dysfunction or irreversible right heart failure, and avoid further intervention for the recurrence of TR.

The present analysis has some limitations. First, this is a small retrospective study with a limited sample size, which reduced the statistical power and generalizability of our results. Larger prospective studies are needed to confirm our findings. Second, propensity score matching was not performed due to the small sample size and the lack of sufficient overlap in the propensity scores between the two groups. Propensity score analysis could further reduce confounding if a larger population is available. Third, this single-center study limits the generalizability of our results to other settings. T-TVR may not benefit patients with more severe right ventricular dysfunction. Moreover, the heterogeneity of our study populations in terms of comorbidities and medications could confound the outcomes of T-TVR. Further multicenter studies are needed to identify the optimal candidates for T-TVR. Fourth, we used TAPSE and RVFAC as echocardiographic measures of RV function. These parameters are widely available and have prognostic value in various cardiac conditions [22]. However, they are not specific markers of RV failure after cardiac surgery and may be affected by RV geometry, loading conditions and ventricular interdependence [23]. Future research may benefit from newer echocardiographic techniques that can provide more accurate assessment of RV function. Finally, our research was restricted to short-term consequences. Another point of contention in this patient population is whether surgery may enhance long-term results in this population, which requires more investigation.

## Conclusion

This study highlights a subgroup of patients with tricuspid regurgitation and reduced RV function after left heart valve replacement. The TAPSE and RVFAC of patients receiving T-TVR had better and faster recovery within the first three months postoperatively than those receiving C-TVR. However, future randomized controlled prospective clinical trials with longer follow-up duration comparing the impact of T-TVR and C-TVR on RV functional recovery and remodeling are needed.

## Data Availability

All data generated or analyzed during this study are included in this published article.

## Abbreviations

TR: Tricuspid regurgitation
RV: Right ventricular
TVR: Tricuspid valve replacement
C-TVR: Conventional tricuspid valve replacement
T-TVR: Total thoracoscopic tricuspid valve replacement
TAPSE: Tricuspid annular plane systolic excursion
RVFAC: Right ventricle fractional area change
VASs: Visual analogue scales
ICU: Intensive care unit
IQR: Interquartile range
CIs: Confidence intervals
CPB: Cardiopulmonary bypass
GEE: Generalized estimating equations
TTVI: Transcatheter tricuspid valve implantation
